# Apical Hypertrophic Cardiomyopathy Presenting With Syncope in a Hispanic Woman: A Report of a Rare Case

**DOI:** 10.7759/cureus.54985

**Published:** 2024-02-26

**Authors:** Yazeed Abu Ruman, Dilesha Kumanayaka, Osama Alkhlaifat, Zaineb Khawar, Noreen Mirza, Addi Suleiman

**Affiliations:** 1 Internal Medicine, Saint Michael's Medical Center, Newark, USA; 2 Cardiology, Saint Michael's Medical Center, Newark, USA

**Keywords:** implantable cardiac defibrillator (icd), icd, non-asian population, hispanic woman, syncope, yamaguchi syndrome, apical hypertrophic cardiomyopathy

## Abstract

Yamaguchi syndrome or apical hypertrophic cardiomyopathy is a rare subtype of non-obstructive hypertrophic cardiomyopathy that is defined as the focused hypertrophy of the left ventricular apex. It is typically seen in Asian populations. Herein, we present a rare case of Yamaguchi syndrome seen in a Hispanic female.

## Introduction

Yamaguchi syndrome is a rare subtype of non-obstructive hypertrophic cardiomyopathy (HCM) concentrated in the left ventricular (LV) apex. First identified in 1975, Yamaguchi syndrome, also known as apical hypertrophic cardiomyopathy (ApHCM), was initially observed in the Asian population, particularly among patients of Japanese descent, with a prevalence of 15-25%. In the United States, specifically, there is a prevalence of approximately 3% of HCM being ApHCM [1]. In predominantly Asian populations, ApHCM accounts for up to 41% of all HCM, highlighting its rarity in non-Asian demographics [2].

ApHCM is a familial disease, and early reports suggest an autosomal dominant inheritance [3]. Upon analyzing multiple genetic markers for HCM, most mutations are found in genes encoding sarcomere proteins [4]. The most common symptom is chest pain; thus, most ApHCM patients present with clinical features mimicking acute coronary syndrome, leading to diagnosis. Diagnosis involves a combination of clinical presentation, physical examination, electrocardiogram findings, and confirmation through imaging modalities [5].

## Case presentation

A 77-year-old Hispanic female with a past medical history of hypertension and hyperlipidemia treated with carvedilol and atorvastatin respectively, with no significant family history of heart disease or sudden cardiac death, presented to the emergency department due to a syncopal episode while walking at home. This was the first time she experienced such an episode. She could not recall any details about how long she was unconscious. A family member found her in the living room. She denied any preceding prodromal symptoms, palpitations or aura, as well as urine incontinence or tongue biting during the event. The patient also denied experiencing any chest pain or shortness of breath. On initial presentation, she was afebrile, with a heart rate of 70 beats per minute, blood pressure of 114/68, a respiratory rate of 12 breaths per minute, and saturation at 94% on room air. Upon physical examination, the patient had normal S1/S2, and no additional murmurs, rubs, or gallops were detected and no carotid bruits on auscultation. There were no focal neurological deficits or orthostatic changes.

Initial laboratory testing revealed normal creatinine kinase (CK-MB) levels, electrolytes, and two sets of troponin. A computerized tomography (CT) scan of the head revealed atrophy and chronic white matter changes, with no acute hemorrhage. The patient was admitted to floors with cardiac monitoring and no arrhythmias were noticed.

**Figure 1:**
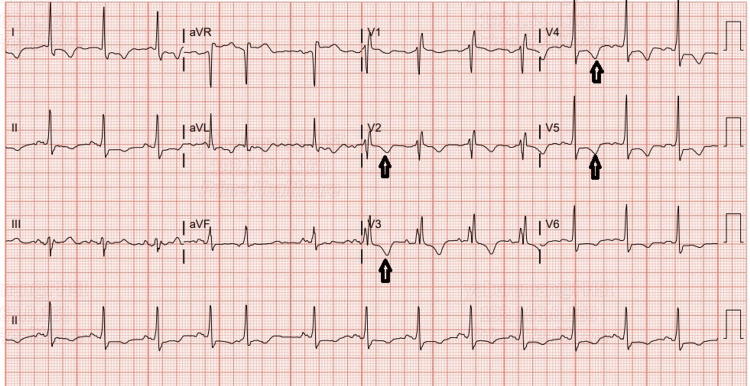
ECG on presentation showing T-wave inversions in the precordial and lateral leads (black arrows). ECG: Electrocardiogram

An electrocardiogram showed normal sinus rhythm with premature atrial complexes, right bundle branch block, and T-wave inversions in the precordial and lateral leads (Figure 1).

**Figure 2:**
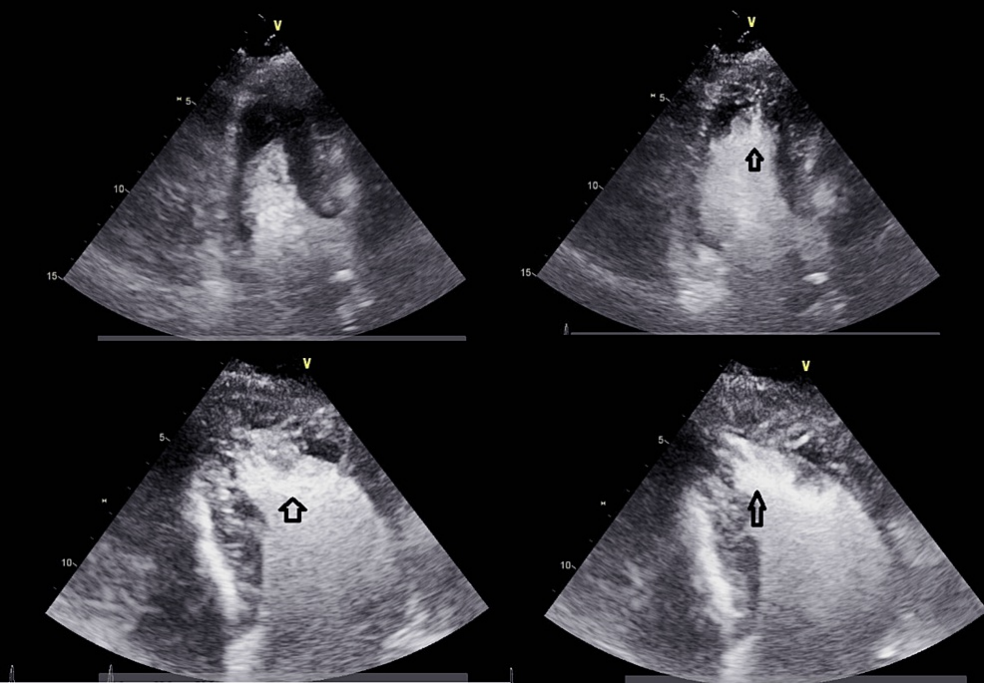
A transthoracic echocardiogram revealed mild to moderate concentric left ventricular hypertrophy with asymmetric thickening of the apical segment (black arrows).

A transthoracic echocardiogram revealed hyperdynamic LV function with an ejection fraction (EF) greater than 70%, grade II pseudo normal filling dynamics, and mild to moderate concentric left ventricular hypertrophy (LVH) with asymmetric thickening of the apical segments consistent with Yamaguchi syndrome (Figure 2).

For secondary prevention, the patient was given the option of receiving an implanted cardioverter-defibrillator (ICD). After a multidisciplinary team conference, the decision was made to proceed with transvenous ICD implantation. Post-implantation chest radiography revealed well-positioned leads. After successful post-implantation device tests, the patient was discharged on carvedilol 3.125 mg twice daily with an outpatient cardiology follow-up.

## Discussion

Yamaguchi syndrome, also identified as ApHCM, represents a distinct variant within the HCM spectrum, primarily affecting the heart's apex. Yamaguchi pioneered the characterization of this syndrome and its ventriculographic features in 1979 [5]. The prevalence of Yamaguchi syndrome varies among different ethnic groups. In the United States, there is a prevalence rate of approximately 3% among the HCM population [6]. However, in Asian populations, ApHCM accounts for up to 41% of all HCM. The heightened prevalence in the Asian population demonstrates the importance of considering ethnic variations when assessing the distribution of Yamaguchi syndrome [2].

The typical age group of ApHCM diagnosis is around 41 years [7] and clinical presentation varies between cases, with approximately half of ApHCM patients being asymptomatic or exhibiting minimal symptoms upon incidental diagnosis. Symptomatic patients may present with syncope, palpitations, anginal chest pain, and shortness of breath. Notably, ApHCM, although associated with a lower mortality rate than other HCM variants, still has the risk of severe complications, including sudden cardiac death [2].

Several indicators contribute to a poor prognosis in ApHCM, including heart failure symptoms, a family history of sudden cardiac death, and a young age at diagnosis. Due to family history being a major contributor to the prognosis of ApHCM, early detection is important for this condition. Guidelines suggest annual screening for first-degree relatives of affected patients with echocardiograms and genetic testing for the disease. Those patients who have a high clinical index of suspicion, a strong family history, and genetic markers should be evaluated with ECG and echocardiography every 3-4 years in adulthood, and 12-18 months during childhood. Genetic testing as a sole screening for ApHCM is unreliable and yields low (approximately 25%) true positive results [1]. These factors underscore the importance of early detection of this condition by screening first-degree relatives of the patients affected with echocardiograms and genetic testing [2].

On physical examinations, patients may have a fourth heart sound (S4) due to impaired LV relaxation. Also, mitral valve abnormalities such as systolic anterior motion and mitral regurgitation are prevalent in approximately half of the ApHCM cases [1]. ECG findings play a crucial role in diagnosing ApHCM. Although giant negative T-waves (≥10 mm) are not pathognomonic, they are observed in around half of the cases, particularly in leads V4-V5. Furthermore, voltage criteria for LVH and T-wave inversions are present in more than 90% of cases [7]. Multimodality imaging, including echocardiography, ventriculography, and cardiac MRI, is very important in diagnosing ApHCM. The "Ace-of-Spades" sign, characterized by apical ventricular wall thickening and cavity narrowing with a normal appearance elsewhere, is a distinctive feature observed through imaging [8].

Diagnosis of ApHCM involves a combination of clinical presentation, examination findings, ECG, and imaging modalities. Treatment strategies mirror those of other HCM variants, with a focus on preventing severe complications and controlling symptoms. Medications such as calcium channel blockers and beta-blockers are employed to regulate heart rate. Also, angiotensin-converting enzyme (ACE) inhibitors are useful as they decrease LV afterload. Despite the importance of adhering to medical treatment, some patients may exhibit syncopal episodes and may be at a high risk of sudden cardiac death, in which cases an ICD is indicated [2].

## Conclusions

The unusual presentation of Yamaguchi syndrome in a 77-year-old Hispanic female, who experienced syncope, deviates from its more typical occurrence in Asian populations. This highlights the need for a comprehensive, multidisciplinary management approach. Echocardiography and other imaging modalities are very important in diagnosing it. The decision to implant an ICD for secondary prevention within the context of ApHCM underscores the crucial importance of preventive measures in reducing the risk of sudden cardiac death.
